# A novel time series analysis approach for prediction of dialysis in critically ill patients using echo-state networks

**DOI:** 10.1186/1472-6947-10-4

**Published:** 2010-01-21

**Authors:** T Verplancke, S Van Looy, K Steurbaut, D Benoit, F De Turck, G De Moor, J Decruyenaere

**Affiliations:** 1 Department of Intensive Care Medicine, Ghent University Hospital, Faculty of Medicine, Ghent University, Ghent, Belgium; 2 Department of Information Technology, Faculty of Engineering, Ghent University, Ghent, Belgium; 3 Department of Medical Informatics and Statistics, Faculty of Medicine, Ghent University, Ghent, Belgium

## Abstract

**Background:**

Echo-state networks (ESN) are part of a group of reservoir computing methods and are basically a form of recurrent artificial neural networks (ANN). These methods can perform classification tasks on time series data. The recurrent ANN of an echo-state network has an 'echo-state' characteristic. This 'echo-state' functions as a fading memory: samples that have been introduced into the network in a further past, are faded away. The echo-state approach for the training of recurrent neural networks was first described by Jaeger H. et al. In clinical medicine, until this moment, no original research articles have been published to examine the use of echo-state networks.

**Methods:**

This study examines the possibility of using an echo-state network for prediction of dialysis in the ICU. Therefore, diuresis values and creatinine levels of the first three days after ICU admission were collected from 830 patients admitted to the intensive care unit (ICU) between May 31th 2003 and November 17th 2007. The outcome parameter was the performance by the echo-state network in predicting the need for dialysis between day 5 and day 10 of ICU admission. Patients with an ICU length of stay <10 days or patients that received dialysis in the first five days of ICU admission were excluded. Performance by the echo-state network was then compared by means of the area under the receiver operating characteristic curve (AUC) with results obtained by two other time series analysis methods by means of a support vector machine (SVM) and a naive Bayes algorithm (NB).

**Results:**

The AUC's in the three developed echo-state networks were 0.822, 0.818, and 0.817. These results were comparable to the results obtained by the SVM and the NB algorithm.

**Conclusions:**

This proof of concept study is the first to evaluate the performance of echo-state networks in an ICU environment. This echo-state network predicted the need for dialysis in ICU patients. The AUC's of the echo-state networks were good and comparable to the performance of other classification algorithms. Moreover, the echo-state network was more easily configured than other time series modeling technologies.

## Background

**Figure 1:**
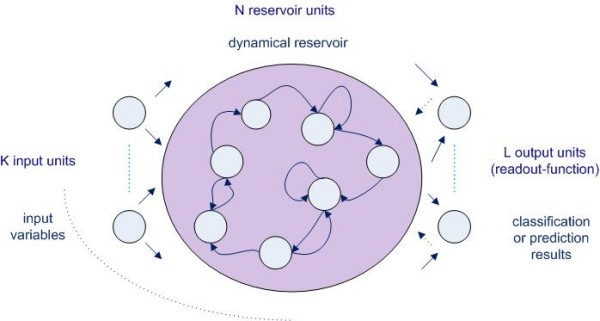
**Basic echo-state network architecture, first described by H. Jaeger **[6], **Dotted lines indicate optional connections within the network topology**

Echo-state networks (ESN), first described by Jaeger H. et al. [1-3], are part of a group of reservoir computing methods and are basically a form of recurrent artificial neural networks (ANN). Modeling of time series in medical databases by classification methods is not easy due to the problem of correlation between the different input variables, also known as the problem of multicollinearity. Analysis of the trend of physiological data however is of vital importance in an ICU environment. Research into techniques that analyze these ICU time series data will become ever more important. The complex modeling of time series can be tackled by using highly specialised tools such as hidden Markov modeling *or *by extracting features from the time series that will be of help to classify unseen data sets which is thus a method of feature extraction. Echo-state networks belong to this *second class *of classification methods. Till this time, no echo-state network applications in clinical research have been published although echo-state network technology for time series prediction has been studied in a variety of engineering applications such as telecommunication [1] and robotics [2], as well as in linguistics to detect grammatical structure [3]. An echo-state network is a 'black box' method since the network does not give clear insight in the parameters of the data model, and thus gives no direct explanatory power. Regression methods with the use of 'penalization', survival analysis with competing risk analysis or functional data analysis are other alternatives for these kinds of time series data sets but will not be discussed in this paper. In an echo-state network, the input variables are applied to a dynamical system called the 'reservoir'. In figure 1, the most general structure of an echo-state network is shown. The reservoir is a recurrent ANN with a large number of units and weighted connections between these units that remain constant. This contrasts with a standard feedforward ANN where these connections vary and are trained via different algorithms, most amply the backpropagation algorithm. The echo-state network however, is trained by modifying the readout function of the network: the readout function of an echo-state network is mapped onto the desired outcome parameter during training till a sufficiently low mean squared error has been reached between the predicted and the real classification data. The training of the readout function of an echo-state network, which is a simple linear function, gives rise to a much more efficient training algorithm than would be the case in a standard feedforward ANN computation. The reservoir of an echo-state network acts as a 'fading memory' (hence the term 'echo-state') and can therefore perform analysis on temporal data such as time series. This could mean a lot of potential future clinical applications since temporal data in the ICU environment are ubiquitous but more difficult to model with statistical regression methods [4,5].

## Methods

**Figure 2:**
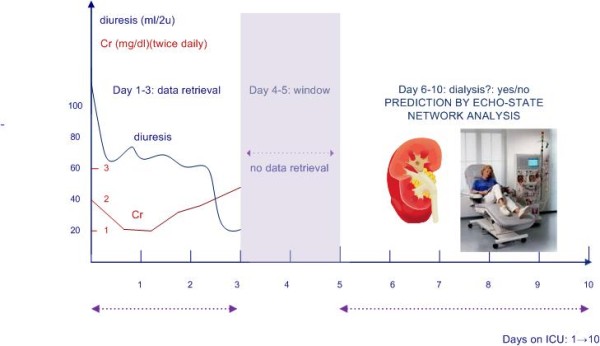
**Graphical outline of the study concept, schematic of timing of data retrieval, data used in the graphic are fictitious and for didactical purposes only**.

The study was approved by the Ethics Committee of the Ghent University Hospital prior to the start of the data retrieval. Informed consent was waived because of the non interventional study design. This study examines the possibility of using an echo-state network for predicting the need for dialysis in the ICU. Moreover, the study compared the performance in prediction obtained by the echo-state network with that obtained by two other time series analysis methods, namely a support vector machine (SVM) and a Naive Bayes classifier (NB) (cf. appendix). To reach these objectives, diuresis and creatinine values were retrieved from the ICU database from a study population consisting of an observational cohort of 916 patients admitted consecutively to the ICU between May 31th 2003 and November 17th 2007, selected from a total of 9752 MICU/SICU patients admitted in this period after application of inclusion/exclusion criteria. Only diuresis and creatinine values of the first three days after ICU admission were retrieved (cf. Figure 2). The outcome parameter in this study was the prediction of dialysis between day 5 and day 10 after ICU admission (cf. Figure 2). 8725 patients with a length of stay (LOS) on the ICU <10 days and 111 patients that received dialysis in the first five days of ICU admission were excluded from analysis. Some further preprocessing of the diuresis and creatinine data was needed: diuresis was only measured in 2 hour intervals while creatinine was measured once, twice or exceptionally three times a day. Hence, the interval between creatinine measurements was larger than the interval between two diuresis measurements. Since the input of time series need to contain measurements over regular time intervals which have to be the same for both parameters, interpolation of the data was the first preprocession step. Furthermore, since the availability of both diuresis and creatinine measurements did not fully overlap, additional preprocessing was at hand. After preprocessing of the data of the 916 patients that had fulfilled the inclusion criteria, 830 patients in total were available with 60 interpolated measurements for both creatinine and diuresis. 62% of these patients were male, mean age of the study population was 58.6 years, total mortality was 17% and mean SAPS II score was 37.2. The echo-state network performance in predicting dialysis was measured by calculating the area under the receiver operating curve (AUC). For comparison, the AUC's for the same prediction problem, obtained by two other time series analysis methods consisting of a support vector machine (SVM) and a naive Bayes (NB) algorithm were calculated. Several parts of the algorithms had a stochastic nature, such as the random initialization of the reservoir weights. Therefore, the ESN, SVM and NB analyses were repeated three times each time using another initialization of the weights in the echo-state network, to see whether or not the variations seen in different analyses were caused by contingent network characteristics. Furthermore, the computational complexity of the three methods will be compared through their required execution times.

### Construction of the echo-state network

**Figure 3:**
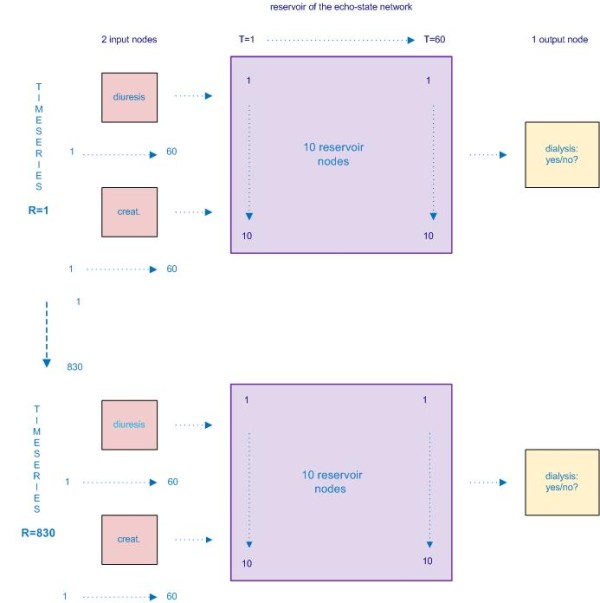
**Schematic of the input, reservoir and the output nodes, number of time points (T1 through T60) and number of time series (i.e. number of patients: R1 through R830)**.

The basic ESN architecture used in this study is shown in figure 1. This basic structure consists of K input units, N reservoir units and L output units. The input variables are presented to the input units, the reservoir units form the dynamical reservoir and the output units represent the classification or prediction results. Remark that - in contrast to a standard feedforward neural network - there are feedback connections to previous hidden layers of the reservoir and that there are loops within single units. First, the different matrices wherein the weights between the units are stored, were configured. These matrices are very important for the functioning of the network, because the echo-state characteristic of the network is dependent upon the mathematical properties of these matrices [6]. Three different matrices are constructed: W^in ^consists of the weights between input and reservoir units, W holds the weights between units of the reservoir, W^out ^the weights between the input, the reservoir and the output units. It is important to note that only the weights of the output function i.e. W^out ^will be mapped onto the desired output. The other matrices (connections/weights) remain constant after initialization, this in contrast with a standard ANN (cf. introduction). Second, the training of the echo-state network was performed by sampling of the teacher data into the network (sampling phase) and calculation of the output weights. Third, after the training phase, the echo-state network was exploited with new unseen data (exploitation phase). During these training and validation phases k-fold cross-validation was used. Cross-validation is a technique wherein the total data set is split into k equally sized parts, called k folds. Each of these k folds are consecutively used for validation of the part of the database 'outside' the k fold, so for validation of the remaining k-1 folds. This procedure is repeated for all of the k folds. The final performance is the total of those measured in each of the k iterations, which thus covers the total amount of available data. The spectral radius of the echo-state network was set at 0.99 [6]. Figure 3 shows the basic scheme of the echo-state network model that was used in this study. The echo-state network for this study consisted of 2 input nodes (one for the diuresis values and one for the creatinine values), 10 reservoir nodes and 1 output node (dialysis: yes/no?). The number of reservoir nodes was selected by analyzing the results of a parameter sweep. Using 10 reservoir nodes resulted in stable results and optimal classification performance. Each node represents a perceptron which is the simplest form of a neural network [7]. The time series presented to the network's two input nodes thus consisted of 60 diuresis values and 60 creatinine values (i.e. 60 points in time from T = 1 to T = 60, cf. Figure 3) for every of the 830 patients included (cf. supra). The time series R_1 _is for patient 1 and time series R_830 _is for the last patient presented to the network. After presentation of the 60 diuresis and creatinine values (i.e. T = 1 till 60) of the first patient, an output weight and the status of the network can be calculated. Indeed, the status of the network at time-point T = 60 is a function of the previously seen data for that patient: the network status is an 'echo' of all previously seen data. Therefore, the output weight together with the status of the network at time-point T = 60 is all that is needed for the training algorithm of the echo-state network. The same is then done for every other patient, till all patients (from R_1 _through R_830_) (Figure 3) have been presented to the network.

### Statistical analysis

The AUC results for the three compared methods (ESN, SVM and NB) were calculated using a 10-fold cross-validation. In each of the different methods, the same folds were used. The AUC results obtained by the echo-state network were then compared with the AUC results of the SVM classifier and the Naive Bayes (NB) algorithm by a non-parametric statistical test [8] within SAS version 9.1.3 (macro %roc). A Dunn-Sidak correction [9] for multiple testing was performed on the obtained p-values.

## Results

In total, 830 patients were retained from the ICU database, after initial assessment of 916 patients for inclusion eligibility and after preprocessing of the data. From these 830 patients, 82 (9.9%) received dialysis and 748 (90.1%) did not receive dialysis between day 5 and day 10 of ICU admission. Table 1 indicates the AUC's in the three consecutive testruns for the prediction of the ESN, SVM and NB networks with two input variables (diuresis and creatinine), the 95% confidence intervals for the developed algorithms and there statistical differences with the echo-state network results. All AUC's demonstrated good discrimination. As shown in Table 1, there were no major differences in AUC's between the different tested methods. Only small statistical differences were seen at the .05 level between the ESN and the NB in testrun 2 and 3, at the advantage of the NB algorithm. It was concluded that ESN, SVM and NB performed well when predicting the need for dialysis in this ICU population (Table 1). So far as computational complexity was concerned, the SVM and NB each required almost 5 hours of computation time, whereas the ESN only required 2 seconds, which was a major advantage for the echo-state network approach.

**Table 1:** AUC's for the three test runs with their respective 95% CI and Dunn-Sidak corrected p-values as statistical difference in comparison with the ESN performance: ESN as reference (ref.) algorithm.

	AUC	95% CI AUC	p-value (ESN = ref.)
*Testrun 1*			
ESN	0.822	0.778-0.865	
SVM	0.831	0.786-0.875	0.238
NB	0.850	0.811-0.890	0.134
*Testrun 2*			
ESN	0.818	0.773-0.864	
SVM	0.833	0.784-0.881	0.356
NB	0.856	0.817-0.894	0.048
*Testrun 3*			
ESN	0.817	0.774-0.861	
SVM	0.833	0.789-0.876	0.093
NB	0.855	0.817-0.894	0.018

## Discussion

This is the first study to investigate the clinical application of echo-state networks for classification in large ICU databases. In general, it is non-trivial to model time series data with classical statistical techniques such as longitudinal data analysis, due to the high degree of correlation within the data. In recent years there has been an evolution towards the development of risk-prediction models that use daily assessment of organ function to evaluate the patient status, and thus incorporate already a certain degree of time dependency [10]. Echo-state networks are specifically designed for the analysis of time series. Other algorithms such as Hidden Markov modeling or dynamic time warping are outside of the scope of this study, but can be suitable alternatives for time series analysis as are methods like functional data analysis and survival analysis methods with consideration of competing risks. The presence of time series in the ICU is ubiquitous and hence the number of possible future ICU applications for this technology are hudge. Echo-state networks have successfully been employed for numerous prediction problems in telecommunication research [1] and robotics [2], as well as in linguistics to detect grammatical structure [3]. Most of these applications come down to prediction of future states of a time series. *In this study however, the basic echo-state network architecture is being adapted so that not only prediction by the network of future states is possible, but finding solutions to classification problems becomes possible too*. It is noticed that the results from the SVM and NB are slightly better than the results obtained by the echo-state network. All AUC's were above 0.8 and clinically acceptable. The time series modeling process in itself was much harder to realize for the SVM and NB, which are not easily configured for time series analysis applications, in contrast to the developing of the echo-state network which is perfectly suitable for time series analysis and therefore relatively easily configured. To be able to input time series in NB and SVM, preprocessing of the data is needed by extracting non-correlated data out of the time series. This preprocessing step needs not to be performed in the echo-state network configuration. The NB and SVM algorithms needed a much longer computation time than the ESN method. These are all clear advantages in favour of the echo-state network approach. It can therefore be concluded that ESN perform well at the task at hand. As a limitation of the study, we can state that no competing risk analysis for competing events (e.g. discharge, death, dialysis before day 5) was performed relating to the more general problem of missing data as seen in other survival analysis methods. The results obtained in this study can be considered as a proof of concept for the use of reservoir computing methods in the ICU. It is clear for every clinician working in an ICU environment that possible future applications for this new data modeling method are amply found: there are a vast number of continuously monitored physiological variables retrieved at the bedside that have time series characteristics. Just to name a few, haemodynamic parameters, ventilatory settings and consecutively retrieved blood samples, are all potential candidates for time series analysis through an echo-state network approach in the ICU. Till now, most of the dynamical and thus time-dependent features of these patient variables were lossed during the modeling process of ICU databases, in spite of the fact that analysis of the trend of physiological data are of vital importance in an ICU environment. The fact that now and in the near future advanced dynamical modeling capabilities through novel technologies such as these described in this study will become possible in clinical practice, is a thrilling evolution for every clinician caring for the welfare of his patients.

## Conclusion

This proof of concept study evaluated the performance of echo-state networks for the first time in predicting the need for dialysis in an ICU population. The classification performance of the echo-state network was good. Moreover, the echo-state network was easily configured compared to SVM and NB modeling techniques, and the echo-state network needed much less computation time. Since time series data in the ICU are amply available and since the modeling of ICU time series data with regression techniques are more difficult due to the problem of high correlation within the data, the authors state that ESN might contribute to the development of future modeling methods of ICU databases.

## Abbreviations

ANN: Artificial Neural Network; AUC: area under the receiver operating characteristic curve; ESN: Echo-State Network; ICU: Intensive Care Unit; MICU: medical intensive care unit; LOS: Length of Stay; LSN: Liquid-State Network; NB: naive Bayes algorithm; SICU: surgical intensive care unit; SVM: support vector machine

## Competing interests

The authors declare that they have no competing interests.

## Authors' contributions

JD, FDT were responsible for the study design and they assume overall responsibility. The data acquisition was performed by KS. Literature research was performed by TV and JD. Data preprocessing was performed by JD and SVL. Development and configuration of the ESN and the ANN were done by SVL. DB and TV performed the statistical analysis. All authors were responsible for the interpretation of data. TV drafted the manuscript. All authors read and approved the final manuscript.

## Appendix

### a. SVM

The heuristic behind the SVM algorithm is quite different from that of the commonly used logistic regression modeling for prediction. This latter approach is the golden standard for prognostic modeling in the ICU and is best known by clinicians. The LR algorithm uses a weighted least squares algorithm, i.e. the prediction is based on construction of a regression line as the best fit through the data points by minimizing a weighted sum of the squared distances to the fitted regression line. SVM, in contrast, tries to model the input variables by finding the separating boundary - called hyperplane - to reach classification of the input variables: if no separation is possible within a high number of input variables, the SVM algorithm still finds a separation boundary for classification by mathematically transforming the input variables and thereby increasing the dimensionality of the input variable space. The general term for a separating straight line in a high-dimensional space is a hyperplane. Moreover, statistical learning theory predicts that the SVM algorithm will find the hyperplane with the maximum-margin to the nearest data point on either side of the hyperplane.

### b. Naive Bayes algorithm

Bayesian theory and Bayesian probability are named after Thomas Bayes, a British eighteenth century mathematician. Bayesian logic combines the result of a test for a particular patient with a pre-test probability (of the population), to forecast or determine the chance of finding a disease: clinicians intuitively combine these two probabilities routinely. Bayesian theory suggests that Bayes' theorem can be used as a rule to infer or update the degree of 'belief' in light of new information (hence the name 'belief networks'). Bayesian networks can be seen as an alternative to logistic regression models where statistical dependence or independence between different variables are explicitly formulated and not hidden in the regression coefficients as in logistic regression. In a naive Bayes network, as used in this study, there are no dependencies between the different feature variables, they are thus considered to be conditionally independent, hence the term 'naive'. A nice example of the applicability in classification problems of these naive Bayesian networks is the article by Price et al. for the classification of cercival cancer patients [11].

## Pre-publication history

The pre-publication history for this paper can be accessed here:

http://www.biomedcentral.com/1472-6947/10/4/prepub
